# Characterization of a stretch-activated potassium channel in chondrocytes

**DOI:** 10.1002/jcp.22075

**Published:** 2010-05

**Authors:** Ali Mobasheri, Rebecca Lewis, Judith EJ Maxwell, Claire Hill, Matthew Womack, Richard Barrett-Jolley

**Affiliations:** 1 Musculoskeletal Research Group, Division of Veterinary Medicine, Faculty of Medicine and Health Sciences, University of NottinghamLeicestershire, United Kingdom; 2 Ion Channel Research Group, Department of Comparative Molecular Medicine, Faculty of Health and Life Sciences, University of LiverpoolLiverpool, Merseyside, United Kingdom

## Abstract

Chondrocytes possess the capacity to transduce load-induced mechanical stimuli into electrochemical signals. The aim of this study was to functionally characterize an ion channel activated in response to membrane stretch in isolated primary equine chondrocytes. We used patch-clamp electrophysiology to functionally characterize this channel and immunohistochemistry to examine its distribution in articular cartilage. In cell-attached patch experiments, the application of negative pressures to the patch pipette (in the range of 20–200 mmHg) activated ion channel currents in six of seven patches. The mean activated current was 45.9 ± 1.1 pA (*n* = 4) at a membrane potential of 33 mV (cell surface area approximately 240 µm^2^). The mean slope conductance of the principal single channels resolved within the total stretch-activated current was 118 ± 19 pS (*n* = 6), and reversed near the theoretical potassium equilibrium potential, E_K+_, suggesting it was a high-conductance potassium channel. Activation of these high-conductance potassium channels was inhibited by extracellular TEA (K_d_ approx. 900 µM) and iberiotoxin (K_d_ approx. 40 nM). This suggests that the current was largely carried by BK-like potassium (MaxiK) channels. To further characterize these BK-like channels, we used inside-out patches of chondrocyte membrane: we found these channels to be activated by elevation in bath calcium concentration. Immunohistochemical staining of equine cartilage samples with polyclonal antibodies to the α1- and β1-subunits of the BK channel revealed positive immunoreactivity for both subunits in superficial zone chondrocytes. These experiments support the hypothesis that functional BK channels are present in chondrocytes and may be involved in mechanotransduction and chemotransduction.

Chondrocytes play a critical role in the synthesis, maintenance, and degradation of extracellular matrix (ECM) macromolecules in load-bearing synovial joints (Archer and Francis-West, 2003; Huber et al., 2000). Recent studies suggest that these functions are modulated by ion channels (Mouw et al., 2007; Wohlrab et al., 2001, 2004). Furthermore, modulation of chondrocyte ion channels by inflammatory mediators may be important in the progression of disease (Sutton et al., 2009). Chondrocytes are exquisitely sensitive to mechanical load and their metabolism is acutely influenced by dynamic changes in the physicochemical environment of articular cartilage (Mobasheri et al., 1998; Lee et al., 2000). Although mechanical load is an important regulator of chondrocyte metabolic activity, the mechanisms of this electro-mechanical coupling are poorly understood (Urban, 1994, 2000). Cartilage responds to load-induced deformation with electrical changes in both the ECM and within the chondrocytes themselves (Lee et al., 2000; Lee and Knight, 2004). Recent studies have provided evidence for hydrostatic and mechanically induced changes in membrane potential of articular chondrocytes under load (Wright et al., 1996; Sanchez and Wilkins, 2004). The deformation of the chondrocyte membrane is thought to be one of several modes of mechanotransduction pathways involved in sensing and responding to changes in mechanical load (Guilak, 1995; Guilak et al., 1995; Knight et al., 1998). Thus, load-induced changes in the chondrocyte membrane, including membrane stretch, are likely to play a key role in the signal-transduction cascades associated with chondrocyte mechanotransduction. The open probability of stretch-activated ion channels generally increases in response to mechanical deformation of the plasma membrane (Sachs and Sokabe, 1990). Although very little is known about chondrocyte stretch-activated ion channels and the macromolecular complexes in which they function, it is thought that they may be linked to the cytoskeleton via β1-integrins (Mobasheri et al., 2002). This may be responsible for their gating by transmitting extracellular physical forces of stretch or pressure to the channels, causing them to undergo a conformational change (Mobasheri et al., 2002). Activation of these ion channels may lead to changes in cell activity via alteration of the resting membrane potential (Mobasheri et al., 2002.) This is supported by studies using ion channel blockers that disrupt the process of mechanotransduction (Wu and Chen, 2000; Mouw et al., 2007). Other studies have suggested that the activation of ion channels may allow the efflux of sufficient ions to drive a decrease in cell volume (regulatory volume decrease) (Hall et al., 1996). The identity of these channels has, however, remained unknown. Information available on the NCBI AceView database suggests that full-length cDNA clones encoding large-conductance (BK-like, MaxiK channels) calcium-activated potassium channels have been isolated from normal and osteoarthritic human articular cartilage and chondrosarcoma cells. There is also some published information about nonspecific mechanosensitive ion channels (Guilak et al., 1999) and transient receptor potential vanilloid 4 (TRPV4) channels in chondrocytes (Phan et al., 2009). However, thus far nothing is known about large-conductance BK-like channel expression and subunit composition in articular chondrocytes. Given the putative emerging role of potassium channels in a variety of cellular processes, we feel that establishing functional roles for these in mineralized tissues would be a welcome advance in the field. Accordingly, in this study, we propose the hypothesis that stretch-activated current is carried by large-conductance (BK-like, MaxiK channels) calcium-activated potassium channels. We used patch-clamp electrophysiology to functionally identify the principal stretch-activated ion channel in equine articular chondrocytes. We also explored the distribution of the stretch-activated channel in sections of equine articular cartilage using immunohistochemistry.

## Materials and Methods

### Chemicals

Unless otherwise stated, all chemicals used in this study were of molecular biology or ACS grade and supplied by Sigma-Aldrich (Poole, UK).

### Cartilage source

Equine joints were obtained from a local abattoir (Nantwich, Cheshire). Articular cartilage was obtained from the femeropatellar, carpal, and metacarpophalangeal joints of skeletally mature male and female horses (*n* = 6). The study was conducted with local institutional ethical approval, in strict accordance with national guidelines. All the animals used were killed for unrelated clinical reasons.

### Histology and tissue processing

Equine cartilage samples were fixed for 24 h in 10% neutral buffered formalin and decalcified in EDTA for a further 72 h. Full-depth samples were embedded in paraffin wax and processed for routine histological and immunohistochemical staining. Stained slide preparations were examined with a Nikon Eclipse 80i microscope. Normal equine sections were cut (7 µm paraffin sections) and mounted on 3-aminopropyltriethoxysilane (APES) treated slides for subsequent immunohistochemical studies.

### Preparation of isolated chondrocytes

Equine cartilage shavings including both mid and superficial layers, but not full depth, were rinsed with phosphate-buffered saline (PBS) then cut into small slices. These were then incubated overnight with type I collagenase (EC 3.4.24.3 from *Clostridium histolyticum*, approximately 100 collagen digestion units ml^−1^) in serum-free Dulbecco's modified Eagles medium (DMEM) supplemented with 1,000 mg l^−1^ glucose and 1% penicillin/streptomycin solution. The filtered cell suspension was washed three times in fresh DMEM and the cells were grown in monolayer culture with 4% fetal calf serum (FCS) for up to two passages. Electrophysiological studies were carried out using freshly isolated chondrocytes, first expansion, first and second passage equine chondrocytes.

### BK channel antibodies

Polyclonal rabbit antibodies developed against the α- and β-subunits of the BK channel (MaxiK) were obtained from Abcam Plc (Cambridge, UK). The immunizing peptide for the α1-subunit of the BK channel corresponds to amino acid residues 945–961 of the human α1-subunit of the human BK protein (KCNMA1 gene, encoding potassium large-conductance calcium-activated channel, subfamily M, alpha member 1): (945) ELVNDTNVQFLDQDDD (961). The immunizing peptide for the β1-subunit of the BK channel corresponds to amino acid residues 90–103 of the human β1-subunit of the human BK protein (KCNMB1 gene, encoding potassium large-conductance calcium-activated channel, subfamily M, beta member 1): (90) YHTEDTRDQNQQC (103). These two sequences are highly conserved across many mammalian species including mouse, rat, and human.

### BK channel immunohistochemistry

Sections of equine cartilage were probed for BK channel expression by immunohistochemistry essentially as previously described (Mobasheri et al., 2005). Equine cartilage tissue microarrays (TMAs) were prepared as described in two recent studies (Mobasheri et al., 2005, 2007) using an Abcam Tissue Micro Array builder (ab1802; Cambridge, UK). Using this approach, equine cartilage samples were arranged in a 6 × 4 grid array on a single-charged microscope slide. This approach increases the throughput for screening equine cartilage samples for BK channel expression using the immunohistochemical technique (Mobasheri et al., 2004). TMA slides were deparaffinized in xylene for 20 min to remove embedding medium and washed in absolute ethanol for 3 min. The TMAs were gradually rehydrated in a series of alcohol baths (96%, 85%, and 50%) and placed in distilled water for 5 min. Endogenous peroxidase activity was blocked for 1 h in a solution of 97% methanol, 3% hydrogen peroxide, and 0.01% sodium azide. The TMAs were then incubated for 1 h at room temperature in Tris-buffered saline (TBS), containing 1% protease-free bovine serum albumin (BSA) and 0.01% sodium azide to block non-specific antibody binding. Slides were incubated overnight at 4°C with rabbit polyclonal antibodies to α (KCNMA1) and β (KCNMB1) subunits of the BK channel. Antibodies were diluted 1:200 in TBS containing 1% BSA. After 24 h at 4°C, the slides were washed three times for 5 min each in TBS containing 0.05% Tween 20 (TBS-T) before incubation with horseradish peroxidase-labelled polymer conjugated to affinity-purified goat anti-rabbit immunoglobulins (code no. K4010; Dako) for 30 min at room temperature. The sections were washed three times for 5 min in TBS-T before application of liquid DAB+ chromogen (3,3′-diaminobenzidine solution; Dako). The development of the brown-colored reaction was stopped by rinsing in TBS-T. The stained slides were immersed for 5 min in a bath of aqueous hematoxylin (code no. S3309; DakoCytomation) to counterstain cell nuclei. Finally, the slides were washed for 5 min in running water and dehydrated in a series of graded ethanol baths before being rinsed in three xylene baths and mounted in 1,3-diethyl-8-phenylxanthine (BDH Laboratories, Atherstone, UK). Control experiments were performed by omitting the primary antibody from the immunohistochemical procedure.

### Microscopy and image acquisition

Immunostained tissue sections were examined with a Nikon Eclipse 80i microscope. Photomicrographs were digitally captured using a Nikon Digital Sight DS-5M camera and Nikon Eclipsenet image capture software.

### Electrophysiological recording

Shards of coverslips with adherent chondrocytes were transferred to a custom tissue chamber and superfused with an extracellular solution (Table 1). Patch pipettes were fabricated from thick walled (Clarke 1.5 mm, filament borosilicate) capillary glass (Harvard Apparatus) on a Brown-Flaming MP P-80 horizontal puller (Sutter Instrument Co.). Pipette resistances were in the range of 5–12 MOhms. Voltage-clamp control was maintained with an Axon 200B Axopatch amplifier (Axon Instruments, Union City, CA), and data were filtered typically at 2 KHz (as appropriate) and digitized (10 kHz) with a DigiData 1200B interface attached to a PC running the AXGOX suit of Axobasic programs (Barrett-Jolley et al., 2000) and/or WinEDR PC software (Dr. John Dempster, University of Strathclyde).

**TABLE 1:** All junction potentials (V_j_) calculated with JPCalcW, by Prof. P. Barry, University of New South Wales, Australia

	K^+^	Li^+^	Na^+^	Mg^2+^	Ca^2+^	Cl^−^	Sucrose	Glucose	HEPES	EGTA	pH	E_k+_ (mV)	V_j_ (mV)
Cell-attached (bath solution)	115	0	0	1.6	2	123	0	10	10	0	7.4	—	—
Cell-attached (pipette; high K^+^)	116	0	0	1.6	2	123	0	10	10	0	7.4	−7^a^	0^1^
Cell-attached (pipette; low K^+^)	14	110	0	1.6	2	131	0	10	10	0	7.4	−57^1^	−6.1^1^
Inside-out (Bath)	145	0	0	1	—2	145	0	0	10	—2	7.2	—	
Inside-out (pipette)	50	0	90	1	0	142	0	0	10	0	7.4	−25^3^	−2.5^3^
Whole-Cell (pipette)	148	0	4	1	0	148	0	0	10	0.5	7.2	−85^4^	2.1^4^
Hypotonic 134 mOsm (bath solution)^5^	5	0	55^6^	1	2	61	0	0	10	0	7.4	—	
Hypotonic 224 mOsm (bath solution)^5^	5	0	55^6^	1	2	61	90	0	10	0	7.4	—	
Isontonic (bath solution)^5^	5	0	55^6^	1	2	61	180	0	10	0	7.4	—	

1 When used with the standard cell attached “Bath” solution and assuming intracellular K^+^ = 150 mM.

2 Either 200 µM Ca was added or 0.5 mM EGTA as stated in the text.

3 When combined with the standard inside-out “Bath” solution.

4 When matched to either of the whole-cell bath solutions.

5 For whole-cell TEA experiments: TEA was maintained at 10 mM both in the bath and the pipette.

^d^When changing the osmolarity of this system, it was essential not to disturb ionic concentrations, thus the only difference between the isotonic and hypotonic bath solutions is the concentration of sucrose. Thus, an initial low Na^+^ concentration was required.

#### Cell-attached patch experiments

Cell-attached patch was performed with high potassium extracellular solution in the bath and pipette (see Table 1), unless otherwise stated. The high extracellular potassium in the bath (Bath solution, Table 1) largely nullifies the resting membrane potential and allows the membrane potential (V_m_) to be estimated directly from V_p_ (the pipette command potential or holding potential, H_p_). For calculation unitary conductance using all-points amplitude histograms (Fig. 2), patches were chosen with low numbers of active channels. This allows characterization of unitary events.

#### Inside-out patch experiments

Inside-out patches of chondrocyte membranes were drawn from cells superfused with a 145 K^+^ (pseudo-intracellular) bath solution, with either 200 µM Ca^2+^ or 0.5 mM EGTA added. The pipette solution, See Table 1. For cell-attached and inside-out patch figures, outward currents are shown as upward deflections. V_m_ is calculated as −V_p_ + V_j_, where V_j_ is the junction potential (see Table 1).

#### Whole-cell membrane potential (V_m_) measurement

Membrane potentials were recorded in whole-cell current clamp mode using an NPI SEC-05LX amplifier with relatively high resistance patch pipettes (approximately 15 MΩ), since the switch clamp electronics of this amplifier are not affected by series resistance. Patch pipettes were filled with a high KCl “intracellular” solution with 0.5 mM EGTA (see Table 1) and the bath was perfused with “extracellular” solutions of varying osmotic potentials (Table 1). Note that it was viewed important to not change the ionic compositions when exposing cells to changes in osmotic potential. To make this possible, NaCl was kept low at all times, and the only change that took place with changing osmolarity was the concentration of sucrose. In some experiments, 10 mM TEA was included; in these experiments TEA concentrations were also kept constant throughout the duration of the recording.

#### Electrophysiological data analysis

Analysis was performed with WinEDR PC software (John Dempster, University of Strathclyde). K_d_s were calculated by solving the following equation

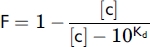
(1)
where F is the fractional current remaining and [c] is the concentration of ligand. K_d_ values are presented with 95% confidence intervals (95% CI) as described previously (Barrett-Jolley et al., 1999; Barrett-Jolley, 2001). Unless otherwise stated, statistical significance was assessed by ANOVA in StatsDirect (Altrincham, Cheshire, UK) with statistical significance defined as *P* ≤ 0.05. Electrophysiological data were fitted in SigmaPlot (Systat Software, Inc. [SSI], San Jose, CA) and Microcal Origin (Northampton, MA). Figures were prepared using SigmaPlot. Values are expressed as mean ± SEM (*n*).

Predicted E_K+_ and E_Cl−_ values given in the text are calculated assuming 150 mM internal potassium and 10 mM internal chloride (Mobasheri et al., 1998). All membrane potentials have been corrected for junction potentials (see Table 1).

All experiments were performed at a slightly elevated room temperature (23–26°C).

## Results

### Cell-attached patch mode

**Fig. 1:**
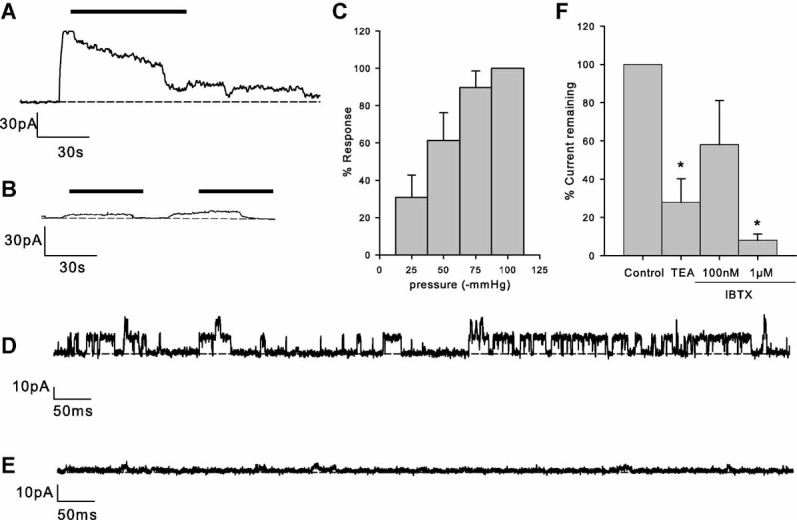
Activation of ion channels by membrane stretch. **A**: Application of suction (as indicated by the solid bar) to the patch pipette of chondrocytes under cell-attached mode of recording increases membrane current. V_m_ = 33 mV. **B**: Similar experiment to “A,” but with 3 mM TEA included in the patch pipette. The suction “response” is greatly reduced. **C**: Increasing negative pressures yield increasingly large activations of current. The y-axis is the percentage current activation of the maximum seen in each particular patch. Data from nine patches. **D**: Single channels (V_m_ = 33 mV) in the “tail” of the suction response for a control experiment similar to that in “A.” **E**: Single channels (V_m_ = 33 mV) in the “tail” of the suction response for an experiment similar to that in “B,” with 3 mM TEA in the patch pipette. **F**: Summary of experiments similar to that shown in A and B. *P ≤ 0.05. IBTX, iberiotoxin.

**Fig. 2:**
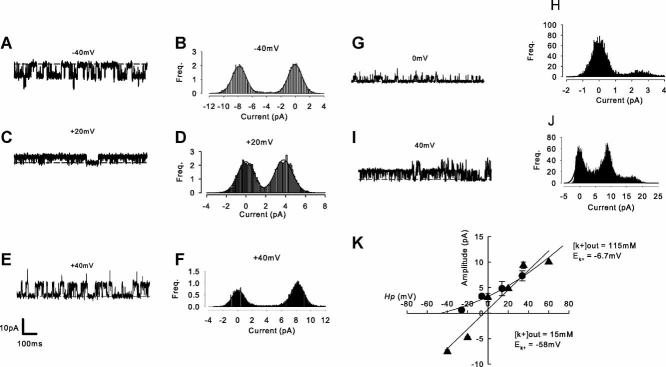
Stretch activates a high-conductance potassium current. **A–F**: cell-attached patch recordings of stretch-activated channel activity with 115 mM pipette [K^+^]. **A**,**C**,**E**: Raw traces at −40, +20, and +40 mV respectively. **B**,**D**,**F**: All-points amplitude histograms for the events shown in corresponding traces A, C, and E. Results from similar experiments are shown in G to J, but with 15 mM [K^+^] in the pipette. K: Mean current-voltage curves from experiments similar to those illustrated in A to J. Triangles: pipette 115 mM (*n* = 6), circles: pipette 15 mM (*n* = 5). The straight line fitted through the 115 mM K^+^ data has slope 192 pS. The 15 mM K^+^ data are fit with the Hodgkin–Katz current equation (Hodgkin and Katz, 1949) assuming the presence of only potassium conductance with P_K+_ 0.4e^−12^ m^3^ s^−1^. Complete solutions and calculated E_K+_ values are described in Table 1.

We applied stretch to chondrocyte membranes by applying negative pressure directly to the patch pipette while recording ion channel activity with the cell-attached mode of the patch-clamp technique. This activated outward current in six of seven patches (Fig. 1). When activated, the mean total current activity was 45.9 ± 1.1 pA at a membrane potential of 33 mV. Under these conditions, the most conspicuous unitary activity was calculated to have a slope conductance of 118 ± 19 pS (*n* = 6, Fig. 2). Activation of current by stretch was significantly inhibited by TEA and by iberiotoxin (Fig. 1) Dissociation constants (K_d_) calculated from Equation 1 were: TEA 0.9 mM (95% CI: 0.5–1.7 mM), *n* = 4. Iberiotoxin 40 nM (95% CI: 28.0–56.9), *n* = 14.

Current-voltage analysis revealed that these channels reversed near to predicted values for E_K+_ (Table 1 and Fig. 2), both with high (116 mM) and low (15 mM) extracellular (pipette solution) potassium concentrations. Slope conductance was 118 ± 19 pS (*n* = 6) with 116 mM extracellular potassium and nonlinear with 15 mM extracellular potassium (note that K^+^ was replaced with Li^+^ since Li^+^ has a low permeability for BK channels (Latorre et al., 1989).

### Isolated patch-clamp experiments

**Fig. 3:**
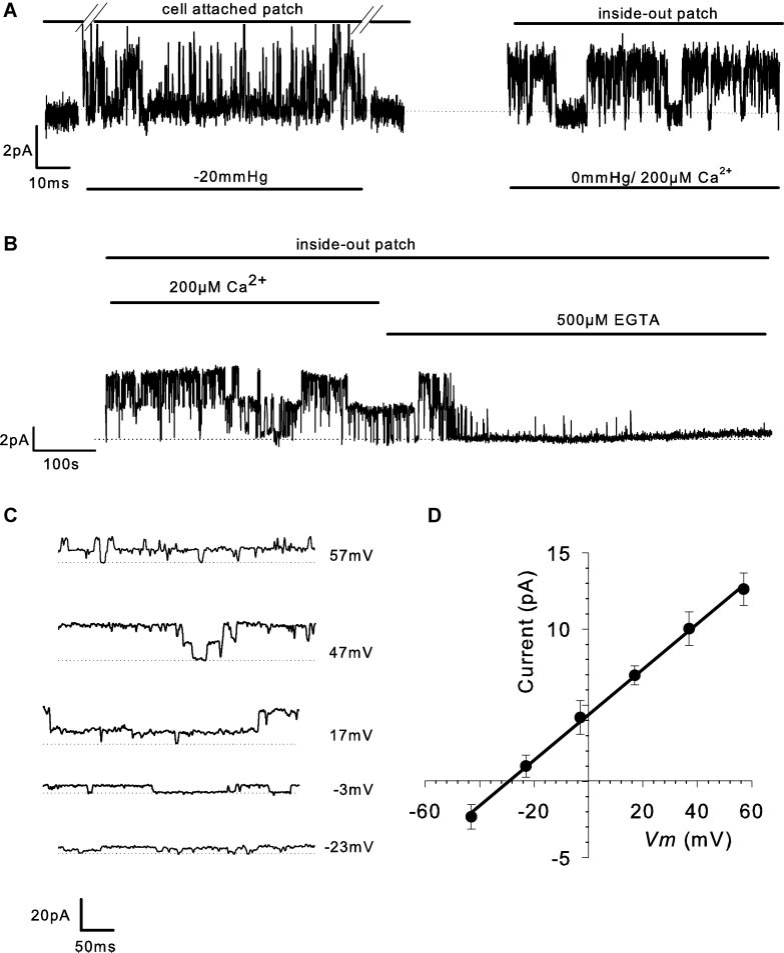
Isolated patch recordings. **A**: Application of negative pressure to the side port of the pipette holder stretches the membrane beneath the patch pipette (cell-attached patch mode) and activates an ion channel current (−3 mV). **B**: Channel activity is still apparent following patch excision (inside-out patch) and is clearly sensitive to 200 µM cytoplasmic calcium or 500 µM EGTA (−3 mV). **C**: Ion channel activity activated as described in A and B (inside-out patch, 200 µM Ca^2+^, but with 145/55 mM K^+^ E_K+_ = 25 mV) at a range of holding potentials. **D**: Current-voltage curves from nine experiments such as that shown in C, in inside-out patch mode. The curve represents a straight line regression with slope 147 pS.

To confirm sensitivity of the channel to cytosolic Ca^2+^, patches with stretch-activated channels were isolated (inside-out patches, Fig. 3) and Ca^2+^ applied to the intracellular face of the membrane (i.e., to the bath solution). In the presence of 200 µM bath Ca^2+^, high levels of channel activity were maintained. In inside-out patch mode, unitary currents again reversed near to E_K+_ (Fig. 3D, and see Table 1); however, as predicted for calcium-activated potassium channels, channel activity ceased when cytosolic calcium was removed (addition of 0.5 mM EGTA).

### Membrane potential (V_m_) measurement experiments

**Fig. 4:**
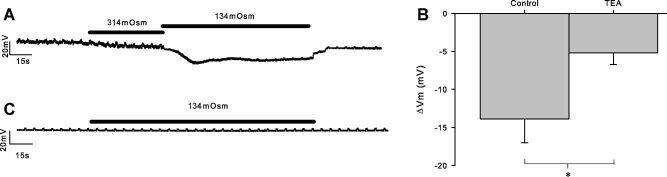
Whole-cell stretch hyperpolarizes chondrocytes. **A**: Application of membrane stretch by means of hypotonic challenge (from 314 to 134 mOsm) hyperpolarizes the membrane. Membrane potential was measured continuously with periodic injections of current to monitor cell integrity. **B**: An equivalent experiment repeated (different cell) in the presence of 10 mM TEA. The hyperpolarization is significantly reduced by the presence of 10 mM TEA (**B**,C). *P ≤ 0.05.

Since we have shown the activation of a significant potassium current (above), one would expect the whole-cell V_m_ to be hyperpolarized by membrane stretch. To apply membrane stretch in this configuration, we applied solutions of decreased osmolarity (Fig. 4). This led to a hyperpolarization of 13.9 ± 3.1 mV (*n* = 9). This hyperpolarization was significantly inhibited by the presence of 10 mM TEA (5.2 ± 1.5 mV, *n* = 6; *P* ≤ 0.05).

### Immunohistochemical distribution of the BK channel

**Fig. 5:**
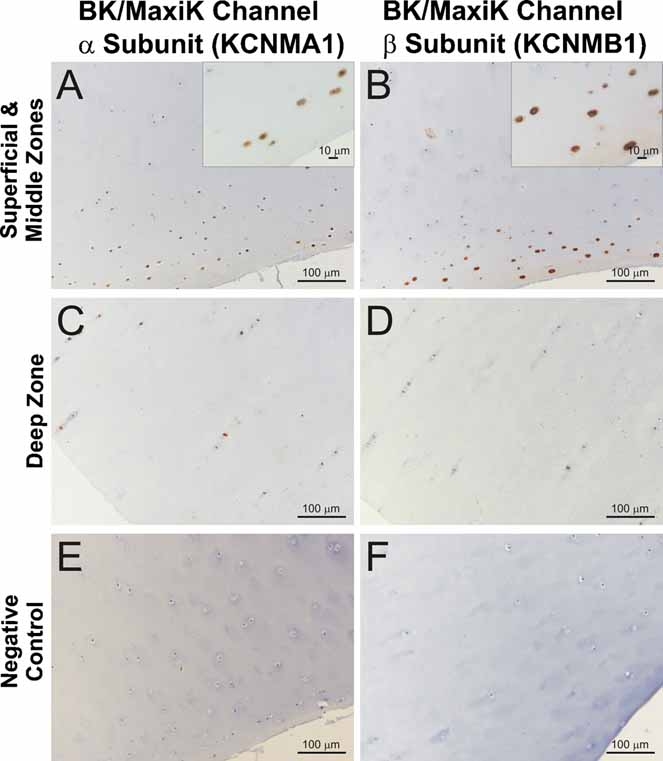
Distribution of the α1- and β1-subunits of the BK channel (KCNMB1 and KCNMNB1) in equine articular cartilage. Immunohistochemical analysis of samples of full-depth equine articular cartilage was carried out using polyclonal antibodies raised against the α1- and β1-subunits of the BK channel. Sections of equine cartilage were immunostained with primary antibodies and horseradish peroxidase-labelled rabbit anti-goat secondary IgG (DakoCytomation). Positive immunoreactivity for both subunits was predominantly observed in superficial zone chondrocytes in normal cartilage. The magnified areas in the insets shown in panels **A** and **B** highlight the chondrocyte-specific immunostaining. Omission of primary antibody from the immunohistochemical procedure served as negative controls. Sections of equine articular cartilage were treated in exactly the same way during the immunohistochemical procedure except that the primary antibody was omitted. Original magnifications of the main panels: 200×. Bars in the main panels represent 100 µm. Bars in the magnified insets shown in panels **A** and **B** represent 10 µm.

The results of the immunohistochemical experiments are summarized in Figure 5.

#### α1-subunit of the BK channel (KCNMNA1)

The α1-subunit of the BK channel was mainly expressed in the superficial zone of macroscopically and microscopically normal articular cartilage from healthy joints (Fig. 5). The immunoreactivity observed for this subunit was much lower in the middle and deep zones of normal cartilage samples.

#### β1-subunit of the BK channel (KCNMNB1)

We observed a similar pattern of immunoreactivity for the β1-subunit of the BK channel in articular cartilage. The β1-subunit was strongly expressed in the superficial zone of normal articular cartilage. As with the α1-subunit, immunoreactivity for the β1-subunit was much lower in the middle and deep zones of normal cartilage samples.

## Discussion

In this study, we used the patch-clamp technique to functionally characterize an ion channel activated in response to mechanically applied membrane stretch in acutely isolated primary equine chondrocytes. These cells hyperpolarize when stretched using a hypotonic challenge applied to the whole cell. This hyperpolarization was greatly reduced by TEA. Having identified functional BK-like calcium-activated potassium channels electrophysiologically, we then employed immunohistochemistry to demonstrate the presence of the α1- and β1-subunits of the BK channel in sections of normal articular cartilage.

We chose the equine chondrocyte model since we have ready access to fresh equine joint tissues. Ideally, one would measure the electrical activity of chondrocytes in vivo or in fresh slices of articular cartilage. However, the presence of a tough ECM of collagens and aggregating proteoglycans makes this a technically impossible prospect. Consequently, most electrophysiological studies are performed with freshly isolated chondrocytes or with cell lines. Work from our own group has established that the primary isolated chondrocytes dedifferentiate to a fibroblastic phenotype after four or more passages in culture (Schulze-Tanzil et al., 2004) and cell lines themselves have the profound limitation that they have little phenotypic homology to primary chondrocytes (see, e.g., Benya and Shaffer, 1982; Gebauer et al., 2005; Schorle et al., 2005). For this reason, we used freshly dissociated chondrocytes and up to second passage cells for patch-clamp electrophysiological studies. This ensures that the cells being studied retain their unique phenotype, but still allows for the application of powerful patch-clamp techniques. Indeed, we have found a strong correlation between our immunohistochemical studies on native tissue and our patch-clamp studies on isolated cells. This has been the case both here and in previous studies (Mobasheri et al., 2005, 2007).

The principal stretch-activated channel we identified in these functional studies had a size (slope conductance), reversal potential, and pharmacology consistent with it being a large calcium-activated potassium channel (BK) (Latorre et al., 1989; Cui et al., 2009). We found the sensitivity to iberiotoxin to be statistically significant but weak. This is interesting because although the BK channel can exist as a standalone six transmembrane α-subunit, complete with potassium conducting pore and Ca^2+^ sensor (Wang and Sigworth, 2009), the presence of a β-subunit modifies many of the channel's functional properties (Salkoff et al., 2006). In particular, low sensitivity to iberiotoxin is highly characteristic of the expression of BK channels consisting of both the α_1_ and β_1_-subunits (Lippiat et al., 2003). This correlated well with our identification of positive immunohistochemical staining for BK channels in normal articular cartilage samples with antibodies both α1- and β1-subunits.

Membrane stretch is a realistic physiological challenge for the chondrocyte and directly linked to changes in matrix production (Urban et al., 1993). Stretch will occur in two contexts. First, when the chondrocyte is deformed by compressive force, the cell passes from a virtually spherical conformation to an approximate ellipsoid (Guilak, 1994; Urban, 1994). For a given volume, an ellipsoid has a greater surface area than a sphere and so membrane stretch is the result. Secondly hypo-osmotic conditions cause cell swelling (Urban et al., 1993), increase cell radius, and consequently increase its surface area. These increases in membrane surface area are likely to be met by stretch rather than the production of a new membrane. This membrane stretch will be limited to a theoretical maximum of approximately 3% at which point it may be expected to rupture (Morris and Homann, 2001). In our experiments, we used two different membrane stretch paradigms, but the results from both were consistent with the opening of potassium channels in response. Both of these approaches have their strengths and weaknesses. The direct membrane stretch (cell-attached patch) experiments allowed us to investigate the situation when neither intracellular or extracellular environments were altered, but this did not allow us to see the effects on the whole cell. Our osmotic challenge experiments required, by definition, alterations of extracellular environment and dialization of the cytosol with the patch-pipette solution. Nevertheless with the whole-cell experiments, we were able to measure the resultant hyperpolarization.

In general terms, there appear to be two possibilities to explain the activation of BK channels by stretch. These could be termed either calcium-dependent or calcium-independent mechanisms. The calcium-dependent hypothesis would require that stretch increases intracellular Ca^2+^ and that this activates the BK channel. Such Ca^2+^ ions could come either from stores (Grandolfo et al., 1998) or from TRP ion channels in the cell membrane (Phan et al., 2009). This is difficult to prove in patch-clamp experiments. In cell-attached patch mode, the intracellular milieu is controlled by the cell itself and, in our whole-cell experiments, Ca^2+^ is buffered partially, but not entirely by EGTA in the patch-pipette solution. More effective calcium buffers are available (BAPTA for example), but with these in the patch-pipette solution there may be brief local rises in Ca^2+^ as, for example, “sparks” (Cheng and Lederer, 2008) that are apparent in many cell types. The calcium-independent hypothesis could involve either direct sensing of stretch by the channel itself or coupling of the channel to other mechanoreceptors such as β_1_-integrins (Mobasheri et al., 2002).

The function of BK channels within the chondrocyte cell membrane is still unknown, but there are a few clear possibilities. First, the BK channel could be acting as an “osmolyte” channel (Hall et al., 1996; Kerrigan and Hall, 2008), since the activation of potassium conductances will allow potassium ion efflux, decreasing intracellular osmotic potential and facilitating regulatory volume decrease. Secondly, it is possible that it is the influence of the BK channel on the membrane potential that is critical, as it is in vascular tissue (Ledoux et al., 2006). In many cell types, BK channels also function as O_2_ sensors (Kemp et al., 2006), and hypoxia is a condition important to the function of chondrocytes (Pfander and Gelse, 2007; Srinivas et al., 2009). BK channels could therefore be involved in coupling O_2_ tension and mechanical pressure to membrane potential. The membrane potential in turn appears to be important for key chondrocyte functions. For example, ion channel blockers, such as SITS and 4-AP, depolarize the membrane potential (Tsuga et al., 2002; Ponce, 2006) and decrease cell proliferation and matrix secretion (Wohlrab et al., 2001, 2004; Mouw et al., 2007). These possibilities will be the subject of future investigations.

## Abbreviations:

BK/MaxiK: large calcium-activated potassium channel
K_d_: dissociation constant
TEA: tetraethylammonium
E_K+_: equilibrium potential for potassium
ECM: extracellular matrix.
